# The Effect of Molecular Weight on the Antimicrobial Activity of Chitosan from *Loligo opalescens* for Food Packaging Applications

**DOI:** 10.3390/md19070384

**Published:** 2021-07-02

**Authors:** Luciana C. Gomes, Sara I. Faria, Jesus Valcarcel, José A. Vázquez, Miguel A. Cerqueira, Lorenzo Pastrana, Ana I. Bourbon, Filipe J. Mergulhão

**Affiliations:** 1 LEPABE—Laboratory for Process Engineering, Environment, Biotechnology and Energy, Faculty of Engineering, University of Porto, Rua Dr. Roberto Frias, 4200-465 Porto, Portugal; luciana.gomes@fe.up.pt (L.C.G.); sisf@fe.up.pt (S.I.F.); 2 Grupo de Reciclado y Valorización de Materiales Residuales (REVAL), Instituto de Investigaciones Marinas (IIM-CSIC), C/Eduardo Cabello, 6, CP36208 Vigo, Spain; jvalcarcel@iim.csic.es (J.V.); jvazquez@iim.csic.es (J.A.V.); 3 International Iberian Nanotechnology Laboratory, Department of Life Sciences, Av. Mestre José Veiga s/n, 4715-330 Braga, Portugal; miguel.cerqueira@inl.int (M.A.C.); lorenzo.pastrana@inl.int (L.P.); ana.bourbon@inl.int (A.I.B.)

**Keywords:** chitin, chitosan, marine waste, antimicrobial activity, poly(lactic acid), active packaging

## Abstract

The growing requirement for sustainable processes has boosted the development of biodegradable plastic-based materials incorporating bioactive compounds obtained from waste, adding value to these products. Chitosan (Ch) is a biopolymer that can be obtained by deacetylation of chitin (found abundantly in waste from the fishery industry) and has valuable properties such as biocompatibility, biodegradability, antimicrobial activity, and easy film-forming ability. This study aimed to produce and characterize poly(lactic acid) (PLA) surfaces coated with β-chitosan and β-chitooligosaccharides from a *Loligo opalescens* pen with different molecular weights for application in the food industry. The PLA films with native and depolymerized Ch were functionalized through plasma oxygen treatment followed by dip-coating, and their physicochemical properties were assessed by Fourier-transform infrared spectroscopy, X-ray diffraction, water contact angle, and scanning electron microscopy. Their antimicrobial properties were assessed against *Escherichia coli* and *Pseudomonas putida*, where Ch-based surfaces reduced the number of biofilm viable, viable but nonculturable, and culturable cells by up to 73%, 74%, and 87%, respectively, compared to PLA. Biofilm growth inhibition was confirmed by confocal laser scanning microscopy. Results suggest that Ch films of higher molecular weight had higher antibiofilm activity under the food storage conditions mimicked in this work, contributing simultaneously to the reuse of marine waste.

## 1. Introduction

One of the major growth segments in the food industry is minimally processed, preservative-free, and ready-to-eat meals and food products [1,2]. As a consequence, the waste from traditional plastic packaging is also increasing (around 4.2% per year) [3] and is considered one of the main factors responsible for short- and long-term environmental pollution [3,4,5]. Another problem facing the food industry is microbial contamination since microorganisms can attach to food and packaging surfaces and form biofilms in a complex and multifaceted process, causing food spoilage, illness, and shelf-life reduction of food products [1,6]. Biofilms are organized communities of microorganisms that attach to a surface and produce extracellular polymeric substances (EPS), which protect them from adverse environmental conditions [6]. These two factors are putting increasing pressure on the food industry to develop new types of antimicrobial packaging materials, mainly based on natural and renewable sources, in order to ensure food safety, quality maintenance, and shelf-life enhancement, as well as to reduce environmental issues caused by non-biodegradable packaging materials [7,8,9].

Among different biopolymers, chitosan (Ch) has received substantial attention from academics and industry for food packaging applications due to its particular physicochemical features, biodegradability, non-toxicity, biocompatibility, good film-forming properties, chemical stability, high reactivity, low cost, and availability in nature [10,11,12]. Chitosan also has intrinsic antioxidant and antimicrobial activities against fungi, molds, yeasts, and Gram-negative and Gram-positive bacteria [13]. As a result of these properties, chitosan was classified as Generally Recognized as Safe by the US FDA in 2001 [14]. However, inherent drawbacks of Ch, including low mechanical and thermal stability and high sensitivity to humidity, have been restricting its industrial application [15]. One strategy to overcome these disadvantages is to combine chitosan with other biopolymers [16] such as the poly(lactic acid) (PLA) used in the present work. PLA is a commonly used polymer for packaging because it has mechanical, thermal, and barrier properties comparable to the most used synthetic plastics, having the advantage of being biodegradable and obtained from renewable sources [17]. Particularly in the last decade, different research groups have focused on the development of PLA/Ch materials [18,19,20,21,22]. Sébastien et al. [18] and Grande and Carvalho [19] obtained composite films by solution mixing and film casting processes. Although the Ch-PLA films produced by Sébastien et al. [18] showed interesting antifungal activity, their heterogeneity and high water sensitivity restrict their usage as food packaging materials [18]. Later, Soares et al. [20] synthesized biodegradable sheets of PLA and coated them with cross-linked chitosan by both spraying and immersion techniques. Nevertheless, the antimicrobial performance of these films was not further evaluated [20]. Bonilla et al. [21] and Chang et al. [22] prepared chitosan–PLA films containing various amounts of chitosan by extrusion and demonstrated their antibacterial activity in refrigerated meat and fish samples, respectively.

The main source of commercial chitosan is chitin, which is the second most abundant polysaccharide on Earth, only preceded by cellulose. Chitin is formed by N-acetylglucosamine units linked by β-(1→4) glycosidic bonds and is commonly sourced from crustacean shells [23], although some molluscs, such as squid, insects [24], and fungi [25], also incorporate this polysaccharide. The disposition of the chitin chains depends on the source; in the case of a squid pen, the source of the chitosan tested in this work, this is parallel (β-chitin), leading to weaker inter- and intra-molecular forces and, as a result, increased solubility and water-absorbing capacity [26]. Weaker forces also carry advantages for the partial deacetylation of N-acetylglucosamine to glucosamine units in chitin to produce chitosan. A sufficient number of glucosamine units enables dissolution in dilute acids due to the protonation of the free amino groups. Furthermore, deacetylation of squid pen β-chitin by alkaline hydrolysis produces more homogeneous chitosan as acetyl groups are more easily accessible than in other sources [27].

In addition to the chemical potential of the chitin/chitosan extracted from the squid pens, their use can help to solve disposal problems for the processing industry and potential environmental impacts. In fact, global squid captures have risen in the last few decades, reaching almost 4 million tonnes in 2015 [28]. Although only representing 5% of all fish, molluscs, and crustaceans captured, squid processing produces a substantial amount of waste. Considering that the yield of edible flesh in squid ranges from 60 to 80% [29], the annual waste generation can be estimated at 0.8 to 1.6 million tonnes and the disposal can be costly in developed countries [30]. Despite the huge potential of chitin and chitosan, currently, they are only employed in a few areas of industrial chemistry, such as cosmetics, textiles, water treatment, and biomedicine [30]. Therefore, a novel concept of shell biorefinery has been suggested on account of the massive potential of chitin valorization. Shell biorefinery consists of the sustainable conversion of chitin into several nitrogen-rich chemicals for pharmaceuticals, cosmetics, textiles, water treatment, household cleansers, soaps, and carbon dioxide sequestration, which benefits both the economy and the environment [30,31,32].

Variations in chitosan’s antibacterial efficacy arise from numerous parameters, including intrinsic factors of chitosan, such as positive charge density, molecular weight (Mw), concentration, and hydrophilic/hydrophobic characteristics [33,34]. In the present work, the effect of Mw on the antimicrobial activity of chitosan is addressed. Although several studies have focused on this parameter, they have generated contradictory results concerning the relation between bactericidal activity and chitosan Mw. Some studies reported that increasing chitosan Mw leads to decreasing chitosan activity against *Escherichia coli*, while others suggested that high Mw chitosan displays greater activity than low Mw chitosan [34].

This study was undertaken to (1) produce β-chitosan and β-chitooligosaccharides from the *Loligo opalescens* pen, (2) develop and characterize PLA surfaces coated with β-chitosan and its derivatives of different Mw, and (3) evaluate the antimicrobial activity of PLA/Ch composite films against *Escherichia coli* and *Pseudomonas putida*, which are bacterial strains present in food processing environments [35,36] and may be responsible for the spoilage of chilled food products [37,38]. This work encompasses the crucial steps for the synthesis and characterization of PLA/Ch films for application in food contact surfaces, from the extraction of chitin and production of chitosan from marine by-products to antimicrobial tests, using innovative combinations of PLA and chitosan and its derivatives.

## 2. Results

### 2.1. Production and Characterization of Chitosan and Derivatives

**Figure 1:**
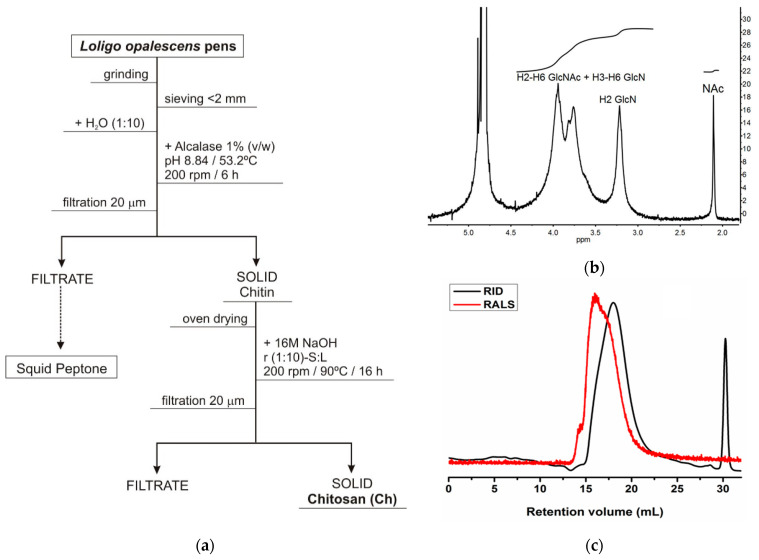
(**a**) Flowchart of chitosan (Ch) production from *Loligo opalescens* squid; (**b**) analysis of Ch by nuclear magnetic resonance (NMR) to calculate the purity and degree of deacetylation; (**c**) eluogram of Ch analyzed by gel permeation chromatography (GPC) for molecular weight determination (RID—refractive index signal, RALS—right angle light scattering signal).

**Figure 2:**
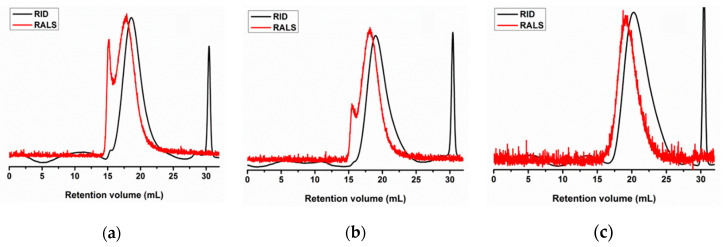
Eluograms of (**a**) β-ChoA, (**b**) β-ChoB, and (**c**) β-ChoC analyzed by gel permeation chromatography (GPC) for molecular weight determination (RID—refractive index signal, RALS—right angle light scattering signal).

**Figure 3:**
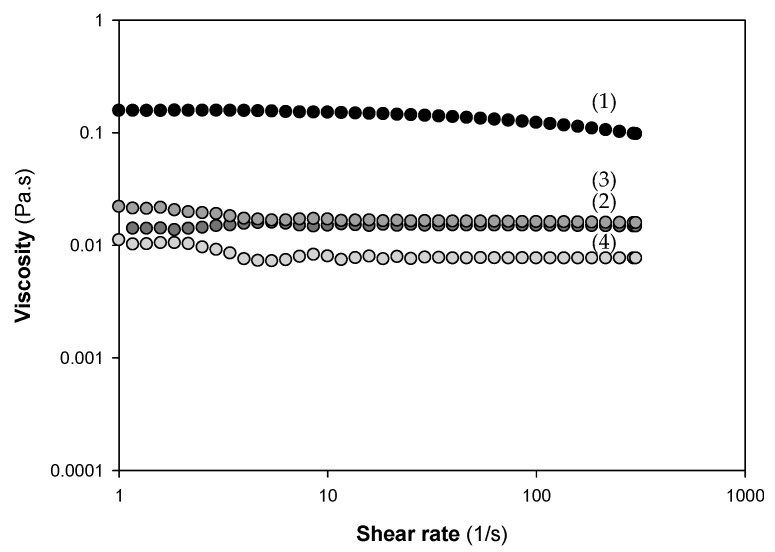
Flow curves of 1% (*w/v*) solutions of chitosan and derivatives used for surface preparation: β-Ch (1), β-ChoA (2), β-ChoB (3), and β-ChoC (4).

Endoskeleton (pen) by-products of the squid species *Loligo opalescens* were initially processed to obtain chitosan by a combination of enzymatic and alkaline treatments, following the optimal conditions defined in a previous work [39]. Alcalase was the enzyme selected for the first step of pen deproteinization to produce chitin, and NaOH was the alkali utilized for the subsequent conversion of chitin into chitosan (Figure 1a). A highly purified β-chitosan (β-Ch) with a 92% of deacetylation degree (Figure 1b) and molecular weights of Mn (number average molecular weight) = 206 kDa/Mw (weight average molecular weight) = 294 kDa was finally recovered (Figure 1c). Based on the protocol of sodium nitrite depolymerization (see Section 4.1), three β-chitooligosaccharides (β-Cho) were produced: (1) a Ch of Mn = 138 kDa/Mw = 186 kDa (β-ChoA, Figure 2a), (2) a Ch of Mn = 84 kDa/Mw = 129 kDa (β-ChoB, Figure 2b), and (3) a Ch of Mn = 37 kDa/Mw = 61 kDa (β-ChoC, Figure 2c).

The rheological behavior of 1% (*w/v*) β-Ch and β-Cho solutions was assessed by evaluating their flow curves at 25 °C (Figure 3). All chitosan solutions revealed a Newtonian behavior, i.e., the viscosity was not dependent on the shear rate. However, it was possible to observe that the β-Ch solution was approximately 10 times more viscous than the three β-Cho samples (β-ChoA, β-ChoB, and β-ChoC).

### 2.2. Characterization of Functionalized Poly(lactic acid) (PLA) Films

#### 2.2.1. Water Contact Angle

**Figure 4:**
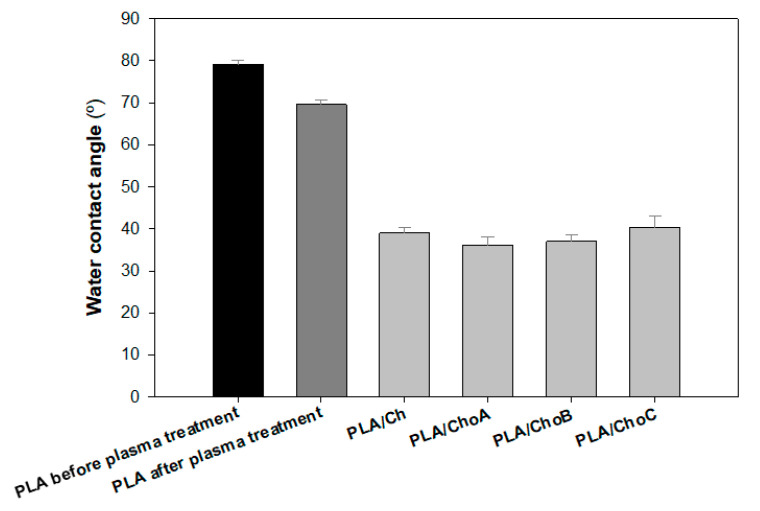
Water contact angles ± standard deviations (SDs) for PLA films before and after oxygen plasma treatment, and PLA/Ch films created by dip-coating method (PLA/Ch, PLA/ChoA, PLA/ChoB, and PLA/ChoC).

Oxygen plasma was applied on the PLA surface to enhance its wettability, adhesion, and biocompatibility [40]. Figure 4 shows the effect of oxygen plasma treatment on the water contact angle of the film. The value of the water contact angle obtained for the PLA before treatment was 79.2 ± 1.1°. A surface with a water contact angle between 0° and 30° can be considered hydrophilic, while a hydrophobic surface is characterized by contact angles over 90° [41]. Therefore, the PLA surface was quite hydrophobic as the initial contact angle for the untreated surface was close to 90°. After the plasma treatment, a significant reduction in the contact angle value was observed. Figure 4 also presents the water contact angles after the deposition of chitosan samples on the PLA surface. PLA films were functionalized with the 1% (*w/v*) β-Ch and β-Cho solutions described above by the dip-coating method. After chitosan deposition, a decrease of approximately 45% in the contact angle of the surfaces was observed compared to the non-functionalized PLA after plasma treatment. This result indicates that chitosan was successfully deposited on the PLA films. Moreover, the type of immobilized chitosan did not influence the wettability of the surfaces since their water contact angles were very similar (around 38°).

#### 2.2.2. Fourier-Transform Infrared Spectroscopy (FTIR) Analysis

**Figure 5:**
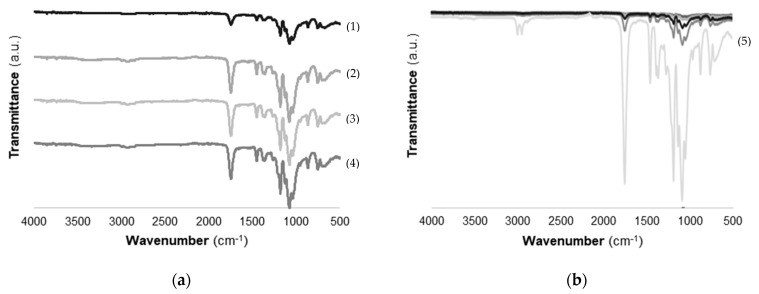
FTIR spectrum of (**a**) different types of chitosan immobilized onto PLA (β-Ch (1), β-ChoA (2), β-ChoB (3), β-ChoC (4)) and (**b**) PLA films (PLA (5)).

FTIR spectra of different types of chitosan immobilized onto the PLA surface are shown in Figure 5. The different types of chitosan deposited onto PLA did not reveal significant differences in FTIR results (Figure 5a). Characteristic bands of chitosan were covered by the presence of bands from the PLA film. The broad _OH stretching absorption band between 3680 and 2750 cm^−1^ and one between 2980 and 2750 cm^−1^ assigned to aliphatic C-H stretching indicated the presence of chitosan on PLA films [42]. Furthermore, a characteristic NH stretching band of chitosan with a maximum at 3350 cm^−1^ was identified on the functionalized PLA/Ch films (Figure 5a).

The FTIR spectrum of PLA films is included in Figure 5b. Results show that characteristic bands of PLA with high-intensity peaks were represented at 1750 cm^−1^ corresponding to CO, at 1188–1090 cm^−1^ corresponding to CO, at 1452–1368 cm^−1^ corresponding to COH, and at 3000 cm^−1^ corresponding to CH. These results are in accordance with those reported by Stoleru et al. [43]. In general, FTIR spectra revealed that surface immobilization of chitosan onto PLA films was successfully achieved.

#### 2.2.3. X-ray Diffraction (XRD)

**Figure 6:**
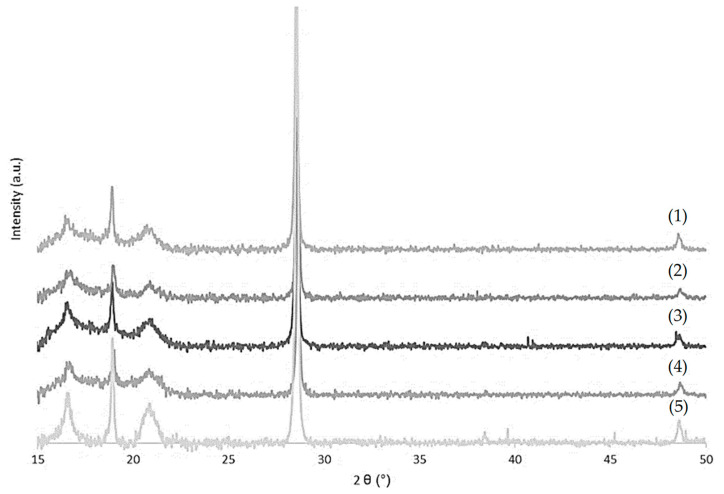
X-ray diffraction (XRD) patterns of different types of chitosan immobilized onto PLA surface (β-ChoC (1), β-ChoB (2), β-ChoA (3), and β-Ch (4)) and of PLA film (5).

The diffraction patterns of PLA films and the effect of different types of chitosan immobilized on PLA are shown in Figure 6. In the case of PLA film, diffraction peaks at 2θ = 16.5°, 20°, and 22° were observed, which corresponded to the characteristic peaks of PLA, indicating a crystalline PLA matrix [44]. An accentuated band at 2θ = 28° suggested the presence of calcium carbonate on PLA films, as described in the literature [45]. The immobilization of chitosan solutions caused a decrease in the intensity peaks at 2θ = 16.5°, 20°, and 22° [46], which confirmed the deposition of chitosan. This modification could indicate a decrease in the crystalline part of PLA/Ch films. It is also possible to conclude that different types of chitosan did not affect the XRD pattern.

#### 2.2.4. Morphological Studies

**Figure 7:**
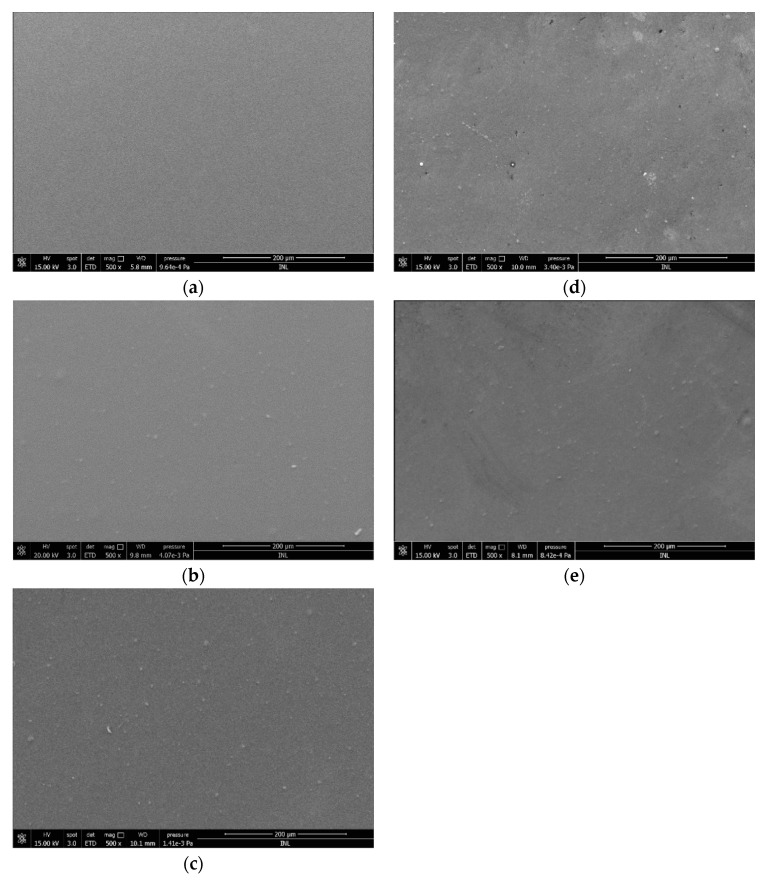
Scanning electron micrographs of (**a**) PLA surface and (**b**–**e**) Ch-based surfaces: (**b**) β-Ch, (**c**) β-ChoA, (**d**) β-ChoB, and (**e**) β-ChoC.

For determination of the surface morphology of the control PLA and Ch-based films, scanning electron microscopy (SEM) analysis was performed. The SEM image corresponding to the PLA film (Figure 7a) reveals a homogeneous surface with a uniform appearance. After the deposition of chitosan onto PLA surfaces, the presence of small particles, which could be unsoluble materials of chitosan, was detected (Figure 7b–e). Although the functionalized surfaces were heterogeneous (Figure 7b–e), no significant differences in PLA/Ch appearance were observed for the different types of chitosan tested.

### 2.3. Antimicrobial Activity of Functionalized PLA Films

The antimicrobial properties of PLA and PLA/Ch surfaces were evaluated in conditions mimicking the storage conditions of packaged food products, namely a short incubation period (1 day), refrigeration temperature (5 °C), and static conditions. Furthermore, two different biofilm-forming bacteria typically associated with the food environment were used: *Escherichia coli* (a model pathogen) and *Pseudomonas putida* (isolated from a salad processing industry) [35,36].

**Figure 8:**
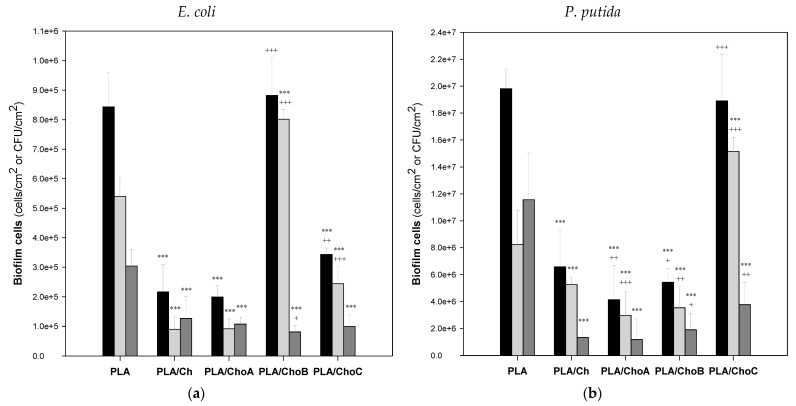
Cellular composition of (**a**) *E. coli* and (**b**) *P. putida* biofilms on PLA and Ch-based surfaces: viable (■), viable but non-culturable (VBNC) (■), and culturable cells (■). Inferential statistics were performed using unpaired *t*-tests or Mann–Whitney tests according to the normality of the variables’ distributions. The means ± SDs for three independent experiments are illustrated. Within the same type of cells, the significance levels were * *p* < 0.05, ** *p* < 0.01, and *** *p* < 0.001 related to PLA (*) and PLA/Ch (^+^).

**Figure 9:**
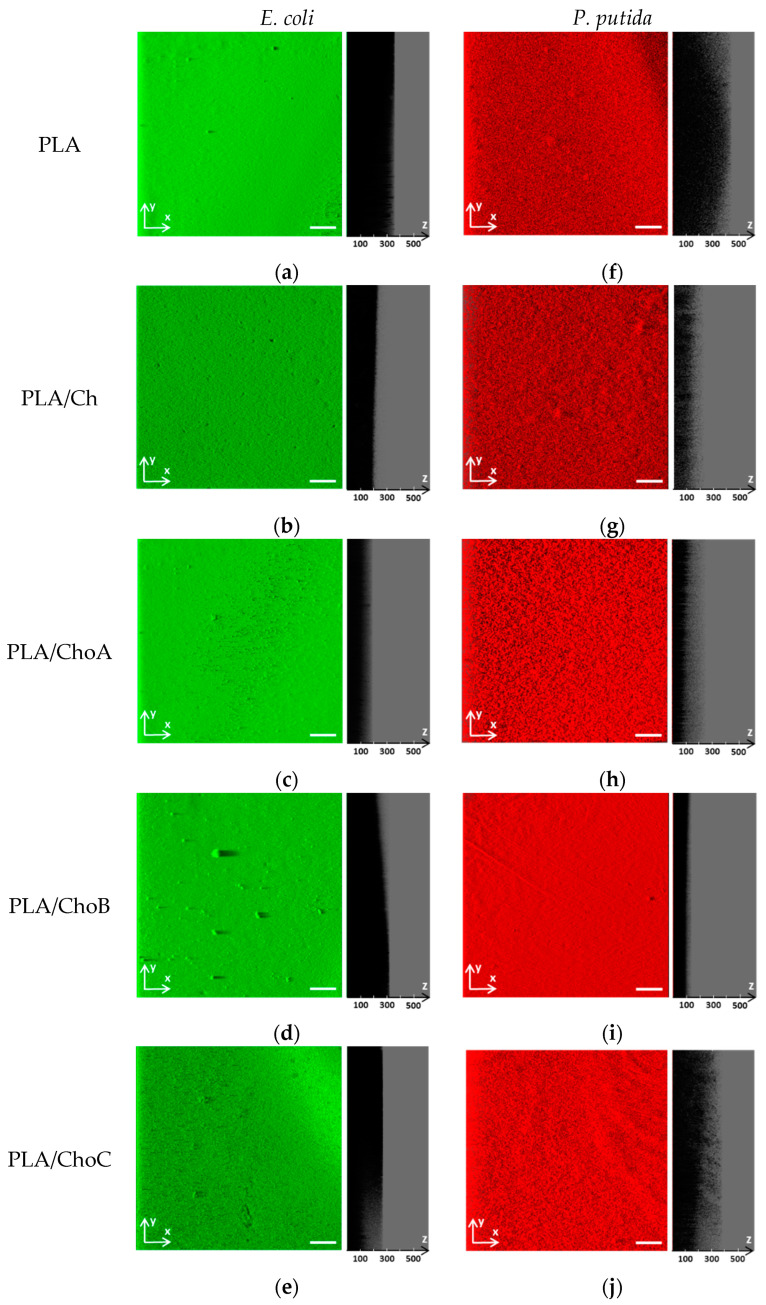
Representative biofilm structures of (**a**–**e**) *E. coli* and (**f**–**j**) *P. putida* on PLA and Ch-based surfaces. These images were obtained from confocal z-stacks using IMARIS software and present an aerial, three-dimensional (3D) view of the biofilms (images on the left). The shadow on the right represents the vertical projection of the biofilm. The white scale bar is 200 μm and the numerical scale indicated in each panel is in μm.

**Figure 10:**
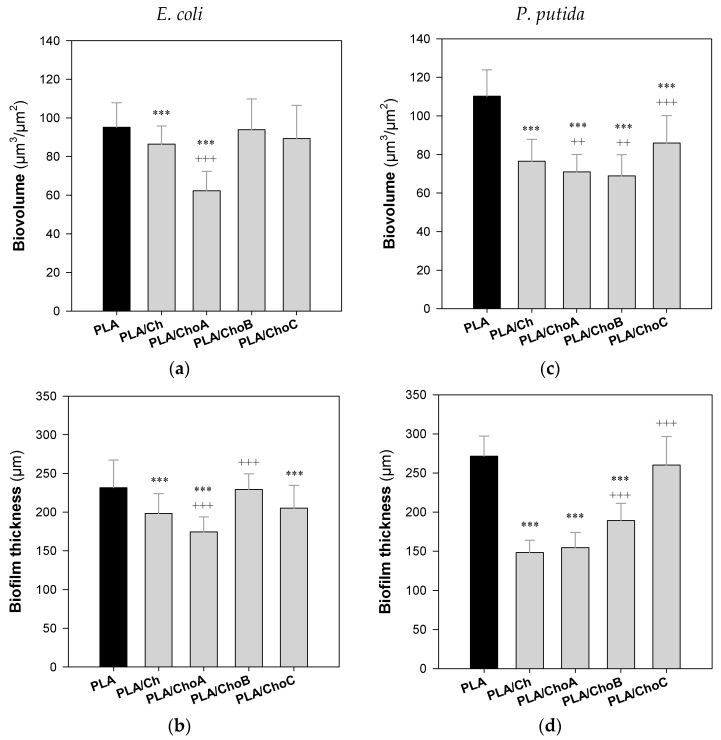
Biovolume and thickness of (**a**,**b**) *E. coli* and (**c**,**d**) *P. putida* biofilms formed on PLA and Ch-based surfaces. The values were obtained from the confocal z-stacks using the COMSTAT2 tool associated with the ImageJ software. Statistical analysis was performed using one-way analysis of variance (ANOVA) and the significance levels were * *p* < 0.05, ** *p* < 0.01, and *** *p* < 0.001 related to PLA (*) and PLA/Ch (^+^).

The cellular composition of *E. coli* and *P. putida* single-species biofilms formed on the control PLA and PLA/Ch films was evaluated by counting viable, viable but non-culturable (VBNC), and culturable cells (Figure 8), whereas the spatial distribution of the biofilms developed by both bacterial strains on the surfaces was analyzed by confocal laser scanning microscopy (CLSM; Figure 9 and Figure 10).

The analysis of *E. coli* biofilm cells (Figure 8a) indicated that there was a significant decrease in the number of all cell types considered (viable, VBNC, and culturable cells) on the chitosan-coated PLA surfaces compared to PLA, except in the case of PLA/ChoB (i.e., the PLA coated by the β-chitooligosaccharide of intermediate molecular weights derived from β-chitosan). Indeed, the biofilms formed on PLA/Ch, PLA/ChoA, and PLA/ChoC surfaces exhibited, on average, 70%, 74%, and 63% fewer *E. coli* viable, VBNC, and culturable cells, respectively, than PLA (*p* < 0.001, Figure 8a). When comparing the antimicrobial efficacy of immobilized native chitosan (PLA/Ch) with its derivatives (PLA/ChoA, PLA/ChoB, and PLA/ChoC, in descending order of molecular weight), it was observed that PLA films coated with depolymerized chitosan had bactericidal behavior similar to PLA films coated with native chitosan (Figure 8a), except for PLA/ChoB.

Regarding the effectiveness of PLA-coated films against *P. putida* biofilm growth (Figure 8b), it was also evident that they were effective in reducing the number of viable, VBNC, and culturable cells by, on average, 73%, 52%, and 87%, respectively. These percentages exclude the cell numbers of the PLA/ChoC surface (i.e., the PLA coated by the β-chitooligosaccharide of higher Mw). Despite having a smaller number of culturable cells than PLA (67%, *p* < 0.001), this coating showed equal or higher numbers of the remaining types of cells.

Looking at the results of the two bacterial strains together (Figure 8a,b), they suggest that the surfaces with the greatest antimicrobial activity were those coated with the native β-chitosan (Mn = 206 kDa/Mw = 294 kDa) and the depolymerized β-chitosan of the highest molecular weight (ChoA, chitosan of Mn = 138 kDa/Mw = 186 kDa). On the other hand, no linear relationship was found between the molecular weight of the tested chitosans obtained from the *Loligo opalescens* pen and their antimicrobial performance under the conditions tested in this study.

The effect of immobilized chitosan and its derivatives against *E. coli* and *P. putida* biofilm formation was also analyzed by CLSM. Both bacteria formed dense and thick biofilms, regardless of the tested surface (Figure 9). Nevertheless, microscopic images revealed that, in general, *E. coli* and *P. putida* biofilms grown on uncoated PLA surfaces were thicker than those developed on PLA films coated with chitosan, which may be related to its antimicrobial activity (Figure 8). These differences in biofilm thickness were particularly evident for PLA/Ch and PLA/ChoA surfaces in the case of *E. coli* (Figure 9b,c), and for PLA/Ch, PLA/ChoA, and PLA/ChoB films with *P. putida* (shadow projection on the right of Figure 9g–i). Furthermore, quantitative data showed a decrease of up to 26% in the average thickness of *E. coli* biofilms formed on PLA/Ch and PLA/ChoA surfaces (shadow projection on the right of Figure 9b), whereas *P. putida* biofilms developed on the same surfaces had approximately 44% and 36% less thickness and biovolume, respectively, than those grown on PLA (shadow projection on the right of Figure 9c,d). Thus, besides bactericidal activity, the native chitosan and its higher Mw derivative also prevented biofilm growth.

## 3. Discussion

The demand for bio-based and safer materials is increasing due to the growth of the human population, industrial development, and environmental concerns. Among known biopolymers, chitosan was selected to confer antibacterial properties to poly(lactic acid) surfaces as it presents remarkable characteristics such as non-toxicity, biocompatibility, and biodegradability [10,11,12]. From the particular perspective of food packaging, easy film formation and antimicrobial activity are considered the most important properties of chitosan, which have been extensively studied [5,7,12,14,47].

Poly(lactic acid) is a polyester formed from 100% renewable raw materials and is highly transparent, hence its intensive use in many disposable packaging solutions. One of the strategies to modify PLA is to apply a surface treatment using a plasma process in order to improve the wettability, adhesion, and biocompatibility of this biopolymer. As expected, the wettability of PLA films used in the present study increased after treatment with oxygen plasma. Identical behavior was reported by Jordá-Vilaplana et al. [40], who used atmospheric plasma treatment to improve the adhesion capacity of PLA films.

In this work, chitosan and its depolymerized derivatives were successfully obtained from a *Loligo opalescens* squid pen by-products via a combination of enzymatic and alkaline treatments [39] and immobilized onto PLA films through plasma oxygen treatment followed by dip-coating. In the last few years, a considerable number of studies have been published on the production of new polymeric systems incorporating chitosan. Some studies have focused on the synthesis of PLA/Ch composites by solution mixing and film casting [19,48,49], but others prepared PLA films by extrusion, coated them with a Ch solution, and used crosslinking agents [20].

Most of the commercial chitosan solutions reported in the literature revealed a visco-elastic behavior, i.e., a high dependence of viscosity with shear rate, except for the low-concentration solutions (lower than 0.25–0.5% (*w/v*)) [50,51]. On the contrary, the results here obtained at 1% (*w/v*) of Ch and derivatives showed a Newtonian behavior, i.e. null dependence of viscosity with shear rate. Other authors observed the same flow behavior, with a Newtonian plateau at a low chitosan concentration of 1.7%, but no Newtonian behavior could be observed at higher chitosan concentrations [52], which suggests that the viscosity of chitosan solutions may be affected by other factors, such as the degree of deacetylation, molecular weight, temperature, and pH [53,54,55]. Additionally, the decrease in viscosity with the decrease in molecular weight among β-Ch and β-Cho was in agreement with the data collected by Chattopadhyay and Inamdar [54]. These authors demonstrated that the viscosity of chitosan is influenced by its Mw and that the intrinsic viscosity of different grades of chitosan decreased approximately ten-fold when the viscosity average molecular weight dropped from 285 to 21 [54]. Furthermore, the decrease in viscosity of β-Cho compared to native chitosan can facilitate the formulation of coatings for application in food technology [56,57].

After the synthesis of the PLA/Ch surfaces, they were tested against two bacterial strains (*E. coli* and *P. putida*) in conditions that simulated the short-term food packaging environment (refrigeration without agitation and 1 day of contact with the cell suspensions). To the best of our knowledge, this is one of the very few studies that evaluates the antibiofilm effect of PLA surfaces coated with native chitosan and derivatives without adding any other compounds, such as essential oils or metallic nanoparticles, to reinforce the antimicrobial properties of coatings and extend the shelf-life of packaged food products [58]. In general, the native chitosan surfaces demonstrated bactericidal activity against the Gram-negative bacteria tested. They decreased the number of biofilm viable, viable but nonculturable, and culturable cells by up to 73%, 74%, and 87%, respectively, compared to PLA. Although the exact mechanism of antibacterial activity of chitosan is still unclear, several mechanisms have been proposed. First, the antimicrobial activity of Ch films is dependent on the degree of deacetylation since the presence of charged amino groups on chitosan molecules can disturb the negatively charged phosphoryl groups on the bacterial cell membrane, leading to its degradation followed by cell death [47]. Chitosan may also form an impermeable layer around the bacterial cell and block the exchange of essential solutes between the intra- and extracellular environment, affecting the physiological state of bacteria and, ultimately, causing cell death [59]. This biopolymer can also diffuse through the cell wall, disrupt the cytoplasmic membrane of bacteria and affect its integrity, as well as suppress the synthesis of RNA and proteins by binding to DNA molecules [58,59].

The antimicrobial activity of chitosan and its derivatives relies on numerous intrinsic and extrinsic factors, including pH, microorganism species, degree of deacetylation of Ch, Mw, concentration, hydrophilic/hydrophobic characteristics, etc. [33,34]. In this work, the surfaces with immobilized chitosan showed different bactericidal performance and inhibited biofilm growth differently. Since the water contact angles and SEM images were identical for all Ch-based surfaces, it is believed that the physicochemical properties and morphology of the films did not affect their antibacterial behavior. Therefore, the main parameter influencing this behavior seems to be the molecular weight of chitosan. Several studies have discussed the relation between bactericidal activity and chitosan Mw, although with contradictory results. Some authors indicated that increasing chitosan Mw leads to decreasing chitosan activity against *Escherichia coli*, while others suggested that high Mw chitosan displays greater activity than low Mw chitosan [34,60]. Hirano et al. [61] showed that chitosans with a Mw of 1.5–4.5 kDa exhibited better inhibitory activities than those with a higher Mw (6.5–12.0 kDa). On the other hand, Tokura et al. [60] disclosed that a 9.3 kDa chitosan inhibited the growth of *E. coli*, whereas a 2.2 kDa chitosan increased bacterial growth. Moreover, some authors have shown that intermediate molecular weights are more effective [34,62]. For instance, Li et al. [62] demonstrated that chitosan suppressed *E. coli* growth, but its inhibitory activity differed with Mw (from 3 to 1000 kDa), with the Ch of 50 kDa Mw having the strongest effect. Similar to Li et al. [62], no linear relationship was found between the Mw of the tested chitosans obtained from the *Loligo opalescens* pen and their antimicrobial performance under the tested conditions. Moreover, the results indicate that the surfaces with the greatest antibiofilm activity were those coated with the native β-chitosan and the depolymerized β-chitosan of the highest molecular weight (ChoA). It is possible that the chitosan of higher Mw interacted with the bacterial cells adhered to the substrate and altered cell permeability, resulting in cell lysis [34]. At the same time, the positive charge of Ch given by the functional amino groups (NH_3_^+^) of N-acetylglucosamine units is expected to react electrostatically with the negatively charged biofilm components such as EPS, proteins, and DNA [57,63], which may explain the lower biovolume and thickness of biofilms exposed to surfaces coated with higher Mw chitosan. It is also reported that high Mw water-soluble chitosan and solid chitosan may form an impermeable layer around the cell surface, thus blocking the transport of nutrients into the cell and causing cell lysis [64,65].

Another interesting result of this work is the capacity that the chitosan-based films showed to reduce the number of viable but nonculturable cells in the biofilms formed by both microorganisms when compared to uncoated PLA (up to 74%). The VBNC state is a unique survival strategy of many bacteria in the environment in response to adverse conditions. The foodborne pathogens may enter the VBNC state during food processing operations, such as disinfection, preservation, and low-temperature storage, and represent a threat to food safety and public health as cells in this state are not detectable through conventional food and water testing methods [66,67]. Indeed, VBNC bacteria cannot be cultured on routine microbiological media, but they remain viable and retain virulence. Therefore, this study suggests that replacing PLA films with PLA coated with chitosan may be an interesting solution to eliminate most VBNC cells in the food packaging environment, preventing foodborne infections and increasing food safety. These Ch-based surfaces may also be beneficial in improving the shelf-life of food products, which is dependent on microbial contamination since certain microorganisms can modify the odor, flavor, color, and textural properties of food products [68].

## 4. Materials and Methods

### 4.1. Production and Chemical Characterization of Chitosan and Chitooligosaccharides

Chitosan previously isolated from the pen of *Loligo opalescens* squid [69] was depolymerized by reaction with sodium nitrite [70]. High molecular weight chitosan of 206 kDa (Mn, number average molecular weight) was initially purified by overnight dissolution in 5% (*v/v*) acetic acid at 9 g/L, followed by filtration (FILTER-LAB® ref. 1250, 10–13 µm; Filtros Anoia, S.A., Barcelona, Spain) and precipitation with methanol:25% ammonia in a proportion 1:3 (*v/v*) chitosan solution:methanolic ammonia. The mixture was left at 4 °C for 1 h, then centrifuged in 1 L bottles at 13,261× *g* on a Beckman Coulter Avanti J-25I centrifuge (Beckman Coulter, Inc, Indianapolis, IN, USA). The precipitates were washed three times with water followed by a final acetone wash, dried overnight at 50 °C on a stove, and freeze-dried. Purified chitosan was milled to a fine powder and dissolved overnight in triplicate in 0.05 M HCl at 8 g/L. Depolymerization reactions were carried out under stirring at room temperature by adding the appropriate amounts of a 1.6 g/L sodium nitrite solution to each chitosan solution, according to the following equation [39]:

(1) $$\frac{1}{M_{f}}-\frac{1}{M_{o}}=\frac{n}{m}$$

where *M_f_* is the molecular weight of chitosan after depolymerization, *M_0_* the initial molecular weight of chitosan, *n* the moles of sodium nitrite, and *m* the initial mass of chitosan. After 4 h of reaction, chitosan was precipitated with 5 M NaOH, the solids separated by centrifugation as described above for the purification process, and washed with water until neutrality. Finally, depolymerized samples were freeze-dried and milled to a fine powder.

The degree of acetylation of chitosan was estimated from nuclear magnetic resonance (NMR) experiments. Chitosan samples (7 g/L) were dissolved in 0.056 M deuterated trifluoroacetic acid (TFA-d in D_2_O), and the corresponding 1H NMR spectra were recorded at 400 MHz (Bruker Avance II; Brucker, USA). The degree of acetylation was calculated from the relative integrals of acetyl (N-acetyl and AcOH) and combined H2–H6 protons (GlcN and GlcNAc) [71,72]. Chemical shifts were expressed in ppm with the HOD solvent signal acting as a reference. Mestrenova 10.0 software (Mestrelab Research, S.L., Santiago de Compostela, Spain) was used for spectral processing.

The molecular weight of chitosan samples was determined by gel permeation chromatography (GPC) on an Agilent 1260 system equipped with a quaternary pump, injector, column oven, and refractive index and static dual-angle light scattering detectors. Chitosan was separated with a set of four columns: Novema Precolumn (10 mm, 8 × 50 mm), Novema 30 Å (10 mm, 8 × 300 mm), Novema 1000 Å (10 mm, 8 × 300 mm), and Novema 1000 Å (10 mm, 8 × 300 mm) from Polymer Standards Service, Mainz, Germany. Column oven and light scattering detector were kept at 30 °C and the refractive index detector was maintained at 40 °C. Samples were eluted with 0.15 M ammonium acetate–0.2 M acetic acid (pH 4.5) as mobile phase at 1 mL/min. Chitosan samples were dissolved in the GPC buffer at a concentration of 1 g/L. Detectors were calibrated with a polyethylene oxide standard of 106 kDa and polydispersity index (PDI) of 1.06 (PSS Polymer Standards Service GmbH, Mainz, Germany). Molecular weight of chitosan was estimated using a refractive index increment (dn/dC) value of 0.18 [73]. From this, two types of chitosan size determination were considered: (1) the average molecular weight of the biopolymer (Mw) and (2) the number average molecular weight of the biopolymer (Mn).

### 4.2. Immobilization of Chitosan and Chitooligosaccharides onto Poly(lactic acid) (PLA) Films

Solutions of chitosan (Ch) and three chitooligosaccharides (ChoA, ChoB, and ChoC) at 1% (*w/v*) were immobilized onto poly(lactic acid) (PLA) films (Goodfellow, UK). For the immobilization of chitosan and derivatives onto the surfaces of PLA films, a plasma oxygen treatment (Harrick Plasma, PJS-14-0240) was applied using a moderate intensity for 15 min. After this, PLA films with dimensions of 1 cm × 1 cm were dipped in the different solutions (Ch, ChoA, ChoB, and ChoC) for 15 min and dried with nitrogen for 5 min.

### 4.3. Rheological Measurements of Chitosan Solutions

Flow curves were obtained using a Discovery Hybrid Rheometer (DHR1) from TA Instruments (New Castle, DE, USA) with Peltier temperature set to 25 °C. TRIOS Software (New Castle, DE, USA) was used to control the equipment and to acquire rheological parameters. A stainless-steel cone-plate geometry of 60 mm, with an angle of 2.006° and truncation of 64 μm, was used due to its capability of generating a uniform shear rate across the samples. Steady-state flow curves were obtained working in a controlled-stress mode, over the shear rate range of 1–300 s^−1^. All the samples (Ch, ChoA, ChoB, and ChoC at 1% (*w/v*)) were measured in triplicate.

### 4.4. Surface Characterization

#### 4.4.1. Water Contact Angle

Films’ surface hydrophobicity was evaluated through the measurement of contact angle by sessile drop technique using a DSA 100E drop shape analysis system (Kruss Gmbh, Hamburg, Germany). A water droplet (2 µL) was deposited at different points on the film surface, and afterwards, a digital camera connected to DSA 3 drop shape image analysis software recorded drop images. The image produced was used to calculate the contact angle via circle fitting method [74]. At least 10 measurements were taken per tested film.

#### 4.4.2. Fourier-Transform Infrared Spectroscopy (FTIR)

FTIR spectra of the films were recorded with VERTEX 80v FTIR spectrometer (Bruker, Germany) in the wavelength range 4000–400 cm^−1^ at a resolution of 4 cm^−1^, using Platinum Attenuated Total Reflection mode (ATR) (Bruker, Germany). The absorbance of each FTIR spectrum was normalized between 0 and 1 [75].

#### 4.4.3. X-ray Diffraction (XRD)

XRD was used to investigate the presence and influence of chitosan on the crystalline structure of the polymer matrix. The XRD patterns of PLA films with and without chitosan were determined using a diffractometer (PanAnalytical X Pert PRO MRD system, Malvern, UK). The scanning range varied from 2θ = 10° to 50° [76].

#### 4.4.4. Scanning Electron Microscopy (SEM)

The morphology of the film’s surface was observed using a scanning electron microscope (Quanta 650 FEG, FEI Europe B.V., Eindhoven, The Netherlands) with an accelerating voltage of +5 kV at different magnifications [75]. The samples were cut with a blade and mounted on sample holders with double-sided adhesive and sputtered with a 10 nm layer of gold.

### 4.5. Antimicrobial Activity of Functionalized PLA Films

#### 4.5.1. Bacterial Strains and Culture Conditions

A model food pathogen—*Escherichia coli* SS2 expressing the green fluorescent protein (GFP) (*E. coli* SS2 GFP)—and an industrial isolate from a salad processing plant—*Pseudomonas putida*—were the bacteria chosen for this study [35,36,77]. Stock cultures were maintained at −80 °C in Tryptone Soy Broth (TSB; BioMérieux, Marcy-l’Étoile, France) containing 20% (*v/v*) glycerol. Before each experiment, frozen cells were subcultured twice in TSB at 30 °C with a constant orbital agitation of 120 rpm [36].

#### 4.5.2. Biofilm Formation

Biofilm assays were performed on 12-well plates (VWR International, Carnaxide, Portugal) for 1 day at 5 °C under static conditions in order to mimic the conditions typically found in short-term food packaging. Before each experiment, the PLA film and the PLA-coated surfaces produced as described in Section 4.2 were sterilized by ultraviolet (UV) radiation for 30 min. Then, the sterilized surfaces were placed on the wells and inoculated with 3 mL of an overnight culture of *E. coli* SS2 GFP or *P. putida* in TSB adjusted to an optical density (OD) of 0.01 at 610 nm (1:10 dilution from an initial cell suspension at OD_610_ nm = 0.1). The microplates were then kept at 5 °C for 1 h to promote bacterial attachment to the surface materials [36,77]. After this adhesion step, the wells were emptied and refilled with 3 mL of sterile TSB, and the microplates were incubated to allow biofilm development. Furthermore, 3 mL of TSB was added to the wells containing sterilized surfaces to monitor their sterility throughout the experiments.

Biofilm formation experiments were performed in three independent assays, each one with three technical replicates.

#### 4.5.3. Biofilm Cell Quantification

Biofilm cell suspensions were obtained by dipping each surface in 2 mL 0.85% (*v/v*) NaCl and vortexing for 3 min. Biofilm cell culturability was assessed after serial dilutions in Tryptone Soy Agar plates (TSA; BioMérieux, Marcy-l’Étoile, France) in the case of *P. putida*, and TSA plates supplemented with 0.1 g/L of ampicillin for *E. coli* SS2 GFP. In turn, biofilm viability was evaluated by staining the biofilm suspension with the Live/Dead^®^ BacLight™ Bacterial Viability kit (Invitrogen Life Technologies, Alfagene, Portugal) as previously described [78] and observing it in an epifluorescence microscope (Leica DM LB2; Leica Microsystems, Wetzlar, Germany). A minimum of twenty fields of view was analyzed for each stained sample using the ImageJ software (version 1.52p, U.S. National Institutes of Health, Bethesda, MD, USA) and the number of viable cells was counted. Finally, the number of VBNC cells was determined by subtracting the number of culturable cells from that of viable cells [79]. The number of culturable cells was presented as CFU/cm^2^, whereas the numbers of viable and VBNC cells were expressed as cells/cm^2^.

#### 4.5.4. Confocal Scanning Electron Microscopy (CLSM)

Single-species biofilms of *E. coli* and *P. putida* that developed after 1 day on all tested surfaces were observed using a 10× dry objective (Leica HC PLAN APO CS) in an inverted microscope Leica DMI6000-CS (Leica Microsystems, Wetzlar, Germany). *E. coli* cells were pinpointed from the GFP expression, while *P. putida* biofilms were stained in red with 5 μM SYTO^®^ 61 (Invitrogen Life Technologies, Alfagene, Portugal), a cell-permeant fluorescent nucleic acid marker. For this reason, *E. coli* biofilms were observed with a 488 nm argon laser, whereas *P. putida* biofilm samples were scanned at an excitation wavelength of 633 nm (helium-neon laser) [36]. A minimum of six stacks of horizontal plane images (512 × 512 pixels, corresponding to 1550 × 1550 µm) with a z-step of 1 μm were acquired for each sample. 

Three-dimensional (3D) projections of biofilm structures were reconstructed using the “Easy 3D” tool of IMARIS 9.1 software (Bitplane, Zurich, Switzerland) directly from the CLSM acquisitions. The plug-in COMSTAT2 associated with the ImageJ software was used to determine the biovolume (µm^3^/µm^2^) and biofilm thickness (μm) [80]. 

### 4.6. Statistical Analysis

Descriptive statistics were used to calculate the mean and standard deviations (SDs) for the number of viable, VBNC, and culturable cells (Figure 8), and biovolume and biofilm thickness (Figure 10). Differences in the number of cells in relation to PLA and PLA/Ch (Figure 8) were evaluated using unpaired *t*-tests or Mann–Whitney tests according to the normality of the variables’ distributions. Quantitative parameters obtained from confocal microscopy (Figure 10) were compared using a one-way analysis of variance (ANOVA). All tests were performed with a confidence level of 95% (*p*-values < 0.05). Data analysis was performed using IBM SPSS Statistics version 24.0 for Windows (IBM SPSS, Inc., Chicago, IL, USA).

## 5. Conclusions

The recent increase in sensitivity towards environmental issues arising from plastic packaging has fostered an interest in alternative sustainable packaging materials. Chitosan and its derivatives were efficiently prepared from fishery waste and immobilized onto PLA films. Their antimicrobial effects were demonstrated in the composite films, with a strong reduction in viable, VBNC, and culturable cell counts in *E. coli* and *P. putida* biofilms. Furthermore, the surfaces with the highest antibiofilm activity were those coated with the native Ch and the Cho with the highest Mw. This is the first time that PLA/Ch surfaces have been shown to be able to eliminate most VBNC cells in the food environment, which is a very interesting result given that such cells retain a public health risk. Further research is needed to bring this biopolymer to industrial levels for food packaging applications.

## Data Availability

The data presented in this study are available on request from the corresponding author. The data are not publicly available yet as some data sets are being used for additional publications.
